# The effect direction plot revisited: Application of the 2019 Cochrane Handbook guidance on alternative synthesis methods

**DOI:** 10.1002/jrsm.1458

**Published:** 2020-10-05

**Authors:** Michele Hilton Boon, Hilary Thomson

**Affiliations:** ^1^ MRC/CSO Social and Public Health Sciences Unit University of Glasgow Glasgow UK

**Keywords:** data visualization, effect direction, standardized metric, synthesis, vote counting

## Abstract

Effect direction (evidence to indicate improvement, deterioration, or no change in an outcome) can be used as a standardized metric which enables the synthesis of diverse effect measures in systematic reviews. The effect direction (ED) plot was developed to support the synthesis and visualization of effect direction data. Methods for the ED plot require updating in light of new Cochrane guidance on alternative synthesis methods. To update the ED plot, statistical significance was removed from the algorithm for within‐study synthesis and use of a sign test was considered to examine whether patterns of ED across studies could be due to chance alone. The revised methods were applied to an existing Cochrane review of the health impacts of housing improvements. The revised ED plot provides a method of data visualization in synthesis without meta‐analysis that incorporates information about study characteristics and study quality, using ED as a common metric, without relying on statistical significance to combine outcomes of single studies. The results of sign tests, when appropriate, suggest caution in over‐interpreting apparent patterns in effect direction, especially when the number of included studies is small. The revised ED plot meets the need for alternative methods of synthesis and data visualization when meta‐analysis is not possible, enabling a transparent link between the data and conclusions of a systematic review. ED plots may be particularly useful in reviews that incorporate nonrandomized studies, complex systems approaches, and diverse sources of evidence, due to the variety of study designs and outcomes in such reviews.

## 1 INTRODUCTION

This article describes a revised method for the effect direction plot^1^ in light of recent Cochrane guidance. Effect direction (evidence to indicate improvement, deterioration, or no change in an outcome) can be used as a standardized metric to encompass a wide variety of data in systematic reviews in which standardized effect sizes cannot be obtained for all included studies. Such situations, in which some form of “narrative synthesis” is conducted rather than meta‐analysis, are common^2^, ^3^; approximately half of Cochrane reviews include a narrative approach to synthesis for some or all outcomes.^4^

The effect direction plot was devised as an approach to support synthesis where effect direction is used as the common metric, principally by providing a way of visualizing the data and promoting transparent links between the data and the narrative.^1^ In the 2013 Cochrane review of housing improvements for health, for example, the effect direction plot was implemented to combine and present findings on four key health outcome domains for outcomes that were conceptually similar but measured in disparate ways.^5^ In each individual study, similar outcomes (eg, cough frequency, cough at night, wheeze, lower respiratory symptoms) are combined into a single outcome domain (in this example, “respiratory health”). An overall direction of effect for the outcome domain for each study is calculated using an algorithm based on the proportion of outcomes within an outcome domain that reported statistically significant effects in a given direction. The effect direction for the outcome domain for each study is then visually represented as an upwards, downwards, or bidirectional arrow. The shading of the arrow indicates whether or not the majority of results for the outcome were statistically significant. The size of the arrow represents the sample size. In the housing improvement review, use of the effect direction plot enabled a 10 000 word review of complex interventions to be summarized and visually displayed in a graphic tabulation on a single page.^1^

The 2019 Cochrane Handbook provides a new chapter on synthesizing and presenting findings using methods other than meta‐analysis.^6^ This chapter notes several acceptable alternatives to meta‐analysis, including vote counting based on effect direction. However, it also notes that vote counting by statistical significance, and vote counting based on subjective rules, are unacceptable. The chapter suggests that a sign test can be applied in the synthesis of effect direction to test whether there is any evidence of an effect, or if the true proportion of effects favoring the intervention is 0.5, that is, no better than chance.

The aim of this study was to apply the new Handbook guidance and update the effect direction plot, using as an example a re‐analysis of the results for the intervention of warmth and energy efficiency improvements included in the Cochrane housing improvement review.

## 2 METHODS

The methods of producing effect direction plots have been previously reported.^1^ To update those methods, statistical significance was removed from the visual presentation of the effect direction and from the algorithm for within‐study synthesis of related outcomes. The use of a sign test was explored to provide a statistical test to support the synthesis of effect direction across studies for any single outcome domain. Studies and data from the Cochrane housing improvement review were re‐analyzed by applying the revised algorithm and conducting sign tests for each outcome.

The effect direction plot was then prepared as per the methods originally reported, but with the following changes. Study quality was represented by shading each study row according to its critical appraisal result, adapting the familiar traffic light system by using green shading for high‐quality and amber for moderate‐quality studies. To support implementation, a github repository for the effect direction plot was created at https://github.com/michelehb/effdir/. The first entry in this repository, an Excel spreadsheet (see Supporting Information in Appendix S1), provides a template for entering data for the plot, including drop‐down lists with the necessary characters for the effect direction arrows, conditional formatting to automatically color the rows according to each study's overall risk of bias, and instructions on using and troubleshooting the template. Box 1 describes the updated method step by step.

The sign test is a nonparametric test that uses a binary measure of either a positive or a negative effect to test whether there is sufficient evidence to reject the null hypothesis of an equal number of positive and negative results.^7^ The *P*‐value from a sign test represents the probability of observing the given number of positive and negative results if the null hypothesis were true. To perform the test, we counted the number of positive and negative effect direction arrows for each outcome domain. Studies with inconsistent effect direction for a given outcome domain were excluded from the count as they could not be said to represent either a positive or a negative effect direction. We used GraphPad (https://www.graphpad.com/quickcalcs/binomial1/) to calculate the two‐tailed *P*‐value for each outcome domain.

## 3 RESULTS

**FIGURE 1:**
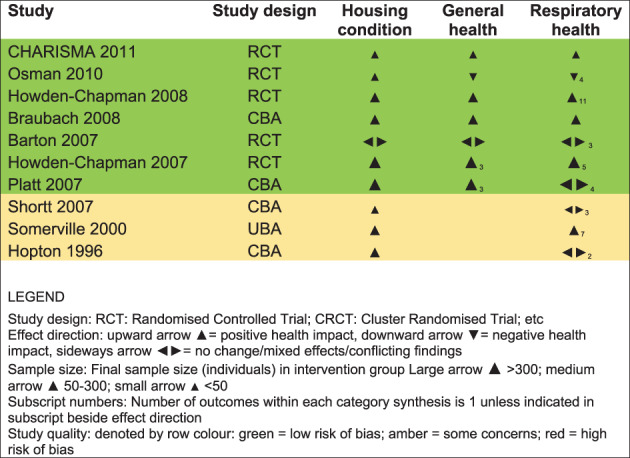
Effect direction plot summarizing direction of health impacts from studies of housing interventions to improve warmth and energy efficiency [Colour figure can be viewed at wileyonlinelibrary.com]

Figure 1 shows the result of applying the updated methods for the effect direction plot to the outcomes of housing condition, general health, and respiratory health reported in the Cochrane review. For housing condition, 9 of the 10 studies reported a positive effect direction, with one study reporting conflicting or unclear effects. The *P*‐value for the sign test for this outcome domain is .0039. For general health, 5 studies reported a positive effect direction, 1 negative, and 1 conflicting/unclear (*P* value for sign test .2188). For respiratory health, 5 studies reported a positive effect direction, 1 negative, and 4 conflicting/unclear (*P* value for sign test .2188). Studies with conflicting or unclear effect direction could not be included in the sign test.

Box 1 **Steps to produce an effect direction plot**

**1. Effect direction synthesis**

Within each study:

1a. Group related outcomes into outcome domains

1b. Count positive and negative effect estimates within each domain

1c. Determine overall effect direction for each domain using the following algorithm:

Where multiple outcomes within a study all report effect in same direction, report that effect direction for the domain

Where direction of effect varies across multiple outcomes within a study:

Report direction of effect where 70% (ie, a clear majority) of outcomes report similar direction

If <70% of outcomes report consistent direction of effect then report no clear effect/conflicting findings ◂▸

**2. Preparation of effect direction plot**

2a. Complete the template (Appendix S1) with study characteristics and outcome domains to be represented.

2b. Within each study‐domain cell, insert arrows to indicate overall effect direction and relative sample size for that study and domain:

Upward arrow ▲ = positive health impact, downward arrow ▼ = negative health impact, sideways arrow ◂▸ = no change/mixed effects/conflicting findings

Set arrow size (small, medium, large) to reflect relative sample size, for example, final sample size (individuals) in intervention group ▲ > 300; ▲ 50‐300; ▲ < 50

If >1 outcome is represented in a study‐domain cell, a subscript number can be placed beside the effect direction arrow to indicate the number of outcomes from the study represented by the arrow

2c. Denote study quality by row color shading: green = high quality/low risk of bias; yellow = moderate/some concerns; red = low quality/high risk of bias.

## 4 DISCUSSION

The updated effect direction plot provides a method of synthesis and visualization of effect direction that removes the reliance on statistical significance to combine multiple similar outcomes from single studies, in line with updated methodological guidance from Cochrane. When meta‐analysis is not possible, alternative methods are needed that support synthesis and data visualization for diverse outcomes while ensuring a transparent link between the data and conclusions of a systematic review.^8^ The revised effect direction plot meets this need and has the potential to be implemented in the majority of public health systematic reviews.

The sign test may provide additional information and aid transparency in the interpretation of the overall pattern of effect direction; however, introducing a sign test to effect direction synthesis raises several issues. First, the power of the sign test may be limited if the review contains a small number of studies, and further limited by the need to discount studies with an unclear effect direction, reducing the number of studies included in the test. Consider a hypothetical example in which a synthesis includes 20 studies, of which 11 have conflicting findings, 8 positive effect direction results, and 1 negative. The sign test will be based on only 9/20 included studies and will arguably misrepresent the synthesis. The effect direction plot has the strength of being able to represent all studies included in a review, but the sign test does not.

Second, well‐recognized caveats about the limitations of *P*‐values and significance testing in judging associations and effects should be kept in mind when reporting syntheses that use the revised effect direction plot.^9^, ^10^ Statements that patterns of effect direction are or are not “statistically significant” should be avoided in line with recent advice.^10^ Instead, results of the sign test might best be used to ensure that the “cognitive algebra”^11^ associated with vote counting remains mindful of uncertainty, and modest in supporting claims regarding intervention effectiveness.

Third, the sign test would only be appropriate if underlying assumptions were met and were appropriately powered. It has been argued that the sign test is inappropriate for synthesis of effect direction because the underlying assumption of a binomial distribution is not met and because it will typically be underpowered in the syntheses where it is likely to be useful.^12^ Additionally, the sign test should not be used in synthesis of effect direction if publication bias is suspected.

We plan to continue to develop this method of visualization and further support its implementation. Future development should consider how sensitive the algorithm is to different methods of addressing within‐study multiplicity of outcomes,^13^ comparison with other methods of visual display of nonstandardized effects,^14^, ^15^ and debates about how to represent and interpret uncertainty.^10^, ^16^ Effect direction as a type of standardized metric merits methodological attention, given the proportion of reviews in which meta‐analysis cannot be undertaken and the need for clear communication of the synthesis of such reviews. Effect direction plots may be particularly useful in reviews that incorporate non‐randomized studies, complex systems approaches, and diverse sources of evidence, due to the variety of study designs and outcomes in such reviews.

## CONFLICT OF INTEREST

The authors reported no conflict of interest.

## Supporting information


**Appendix S1.** Supporting Information

## Data Availability

Data sharing is not applicable to this article as no new data were created or analyzed in this study.
